# Haploinsufficiency in the mitochondrial protein CHCHD4 reduces brain injury in a mouse model of neonatal hypoxia-ischemia

**DOI:** 10.1038/cddis.2017.196

**Published:** 2017-05-11

**Authors:** Yanyan Sun, Tao Li, Cuicui Xie, Yiran Xu, Kai Zhou, Juan Rodriguez, Wei Han, Xiaoyang Wang, Guido Kroemer, Nazanine Modjtahedi, Klas Blomgren, Changlian Zhu

**Affiliations:** 1 Henan Key Laboratory of Child Brain Injury, Department of Pediatrics, The Third Affiliated Hospital of Zhengzhou University, Zhengzhou, China; 2 Center for Brain Repair and Rehabilitation, Institute of Neuroscience and Physiology, Sahlgrenska Academy, University of Gothenburg, Gothenburg, Sweden; 3 Department of Pediatrics, Zhengzhou Children’s Hospital, Zhengzhou, China; 4 Department of Women’s and Children’s Health, Karolinska Institutet, Stockholm, Sweden; 5 Perinatal Center, Institute of Neuroscience and Physiology, Sahlgrenska Academy, University of Gothenburg, Gothenburg, Sweden; 6 INSERM, U1138, Paris, France; 7 Equipe 11 labellisée par la Ligue Nationale contre le Cancer, Centre de Recherche des Cordeliers, Paris, France; 8 Université Paris Descartes/Paris V, Sorbonne Paris Cité, Paris, France; 9 Metabolomics and Cell Biology Platforms, Gustave Roussy Cancer Campus, Villejuif, France; 10 Laboratory of Molecular Radiotherapy, INSERM U1030, Gustave Roussy, Villejuif F-94805, France; 11 Gustave Roussy, Villejuif F-94805, France; 12 Department of Medicine, Université Paris-Saclay, Kremlin-Bicêtre, France; 13 Department of Pediatric Oncology, Karolinska University Hospital, Stockholm, Sweden

## Abstract

Mitochondria contribute to neonatal hypoxic-ischemic brain injury by releasing potentially toxic proteins into the cytosol. CHCHD4 is a mitochondrial intermembrane space protein that plays a major role in the import of intermembrane proteins and physically interacts with apoptosis-inducing factor (AIF). The purpose of this study was to investigate the impact of CHCHD4 haploinsufficiency on mitochondrial function and brain injury after cerebral hypoxia-ischemia (HI) in neonatal mice. CHCHD4^+/−^ and wild-type littermate mouse pups were subjected to unilateral cerebral HI on postnatal day 9. CHCHD4 haploinsufficiency reduced insult-related AIF and superoxide dismutase 2 release from the mitochondria and reduced neuronal cell death. The total brain injury volume was reduced by 21.5% at 3 days and by 31.3% at 4 weeks after HI in CHCHD4^+/−^ mice. However, CHCHD4 haploinsufficiency had no influence on mitochondrial biogenesis, fusion, or fission; neural stem cell proliferation; or neural progenitor cell differentiation. There were no significant changes in the expression or distribution of p53 protein or p53 pathway-related genes under physiological conditions or after HI. These results suggest that CHCHD4 haploinsufficiency afforded persistent neuroprotection related to reduced release of mitochondrial intermembrane space proteins. The CHCHD4-dependent import pathway might thus be a potential therapeutic target for preventing or treating neonatal brain injury.

Perinatal asphyxia-induced cerebral hypoxic-ischemic (HI) brain injury is an important cause of neonatal death and permanent neurological disability in children, especially in developing countries,^1^ and interventions against perinatal brain injury have shown promising results in reducing the incidence of neurological deficits.^2, 3^ However, these treatments are not successful in all cases, and hypothermia is not suitable for very preterm infants,^4, 5^ thus there is a pressing need for a better understanding of the mechanisms of brain injury and for conducting translational studies on how to reduce cell death, increase cell survival, and promote brain regeneration and repair after perinatal brain injury.^6^

Mitochondria are pluripotent organelles with multiple cellular functions, including the regulation of physiological metabolism, stress responses, and cell death, and they play an important role in the process of brain injury after insult.^7^ Mitochondria are key regulators of apoptotic cell death,^8, 9^ and following an insult mitochondria are permeabilized and cell death-related proteins are released from the mitochondria into the cytosol, including cytochrome *c* (Cyt *c*) and apoptosis-inducing factor (AIF), which, respectively, trigger caspase-dependent and caspase-independent apoptotic cell death.^10^ These processes are more prominent in the immature brain than in the adult brain,^11^ and interventions targeting mitochondria have shown promising results against immature brain injury.^12, 13, 14^

AIF is a bifunctional intermembrane space protein that was first identified as a caspase-independent death effector.^15^ Further studies in mice carrying a hypomorphic mutation, yielding 60% AIF protein downregulation, found that AIF plays important roles in cell survival, proliferation, and differentiation by participating in mitochondrial metabolism.^16, 17, 18, 19, 20^ AIF downregulation reduces the abundance of respiratory chain protein complexes, which compromises oxidative phosphorylation.^16, 21^ We previously found that the caspase-independent AIF pathway is involved in perinatal brain injury,^22^ and we subsequently showed that AIF plays a causal role in neuronal cell death and brain injury,^23, 24^ and that it requires the interaction with cyclophilin A to induce chromatin degradation.^25, 26^ Recently, we^21^ and others^27^ identified CHCHD4 as an AIF-interacting protein and found that AIF downregulation is correlated with decreased CHCHD4 protein levels without affecting mRNA transcription. Moreover, CHCHD4 overexpression counteracts the loss of respiratory subunits that is normally observed after depletion of AIF.^21, 27^ These results suggest that CHCHD4 provides a mechanistic link between AIF deficiency and mitochondrial dysfunction.

CHCHD4, which is the human homolog of yeast Mia40, is the key component of the mitochondrial disulfide relay system that regulates the import, folding, and oxidative maturation of a group of small nuclear-encoded, cysteine motif-carrying proteins that participate to a vast panel of activities that include mitochondrial bioenergetics, protein import, intramitochondrial lipid homeostasis, anti-oxidant response, or mitochondrial ultrastructural organization.^28, 29, 30^ CHCHD4, which has the capacity to bind the iron, has recently been revealed to be an indispensable component of the iron–sulfur cluster export machinery, and its depletion results in the accumulation of iron in mitochondria.^31^ It has been reported that CHCHD4 controls mitochondrial Ca^2^+ uptake by triggering the heterodimerization of MICU1 and MICU2, and the subsequent interaction of the dimer with the regulator of the mitochondrial Ca^2+^ uniporter that transfers Ca^2+^ across the inner membrane.^32^ CHCHD4 has also been reported to control HIF-1*α*-dependent hypoxia signaling by regulating oxygen consumption rates.^33^

Downregulation of CHCHD4 results in mitochondrial defects that are similar to those observed in AIF downregulation,^21^ which indicates that CHCHD4 and AIF are epistatic with respect to mitochondrial biogenesis and respiratory function. The observation that the depletion of AIF causes the downregulation of CHCHD4 protein by diminishing its mitochondrial import sheds a new light on the regulation of the CHCHD4-dependent import machinery and reveals AIF as one of its critical components.^21, 29^ Our previous studies have shown that AIF downregulation affords neuroprotection in the neonatal brain after HI,^23, 25^ but it is still unclear if CHCHD4 downregulation has any effect on mitochondrial function and brain injury in the developing brain. The purpose of this study was to evaluate the effects of CHCHD4 downregulation on neonatal HI brain injury and the possible mechanisms behind such effects.

## Results

### CHCHD4 haploinsufficiency reduces neonatal HI brain injury

**Figure 1:**
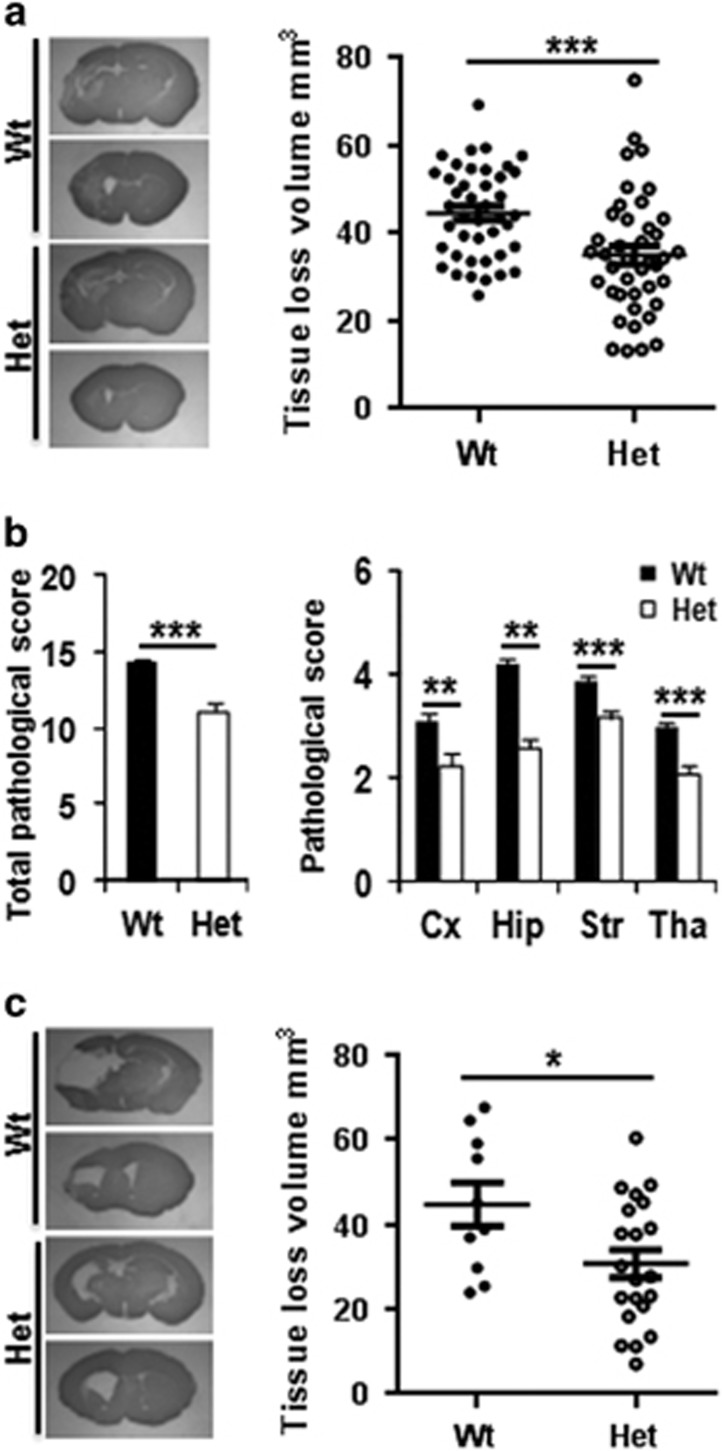
CHCHD4 deficit confers protection against HI brain injury. (**a**) The photomicrographs (left) show representative MAP2 staining obtained 72 h after HI at the dorsal hippocampus and the striatum levels. The graph (right) shows the total tissue loss 72 h after HI (*n*=42 for Wt and *n*=41 for Het). (**b**) The bar graphs show the total pathological score and the pathological scores in the cortex (Cx), hippocampus (Hip), striatum (Str), and thalamus (Tha) in Wt and Het mice at 72 h after HI. (**c**) The microphotographs show representative MAP2 staining at 4 weeks after HI from the dorsal hippocampus and the striatum levels. The graph shows the tissue loss at 4 weeks after HI (*n*=10 for Wt and *n*=21 for Het). *P<0.05, **P<0.01, ***P<0.001

The short-term influence of CHCHD4 downregulation on brain injury was evaluated at 72 h after HI based on the total tissue loss volume (infarction+atrophy) and pathological score (Figures 1a and b). The total tissue loss volume was reduced by 21.5% in CHCHD4^*+/−*^ heterozygous (Het) mice (34.89±2.15 mm^3^, *n*=41) compared to wild-type (Wt) mice (44.47±1.60 mm^3^, *n*=42) (*P*=0.0006) (Figure 1a). Further analysis showed that the brain injury reduction in Het mice included all of the examined brain regions compared with Wt littermates as evaluated by neuropathological scores (Figure 1b). The tissue loss volume was reduced by 26.6% in male Het mice (34.82±3.73 mm^3^, *n*=20) compared with male Wt mice (47.40±2.30 mm^3^, *n*=21) (*P*=0.0061) and by 15.8% in female Het mice (34.96±2.35 mm^3^, *n*=21) compared with female Wt mice (41.54±2.08 mm^3^, *n*=21) (*P*=0.0423). There was no significant difference in tissue loss volume between male and female mice for either group (data not shown). The long-term influence of CHCHD4 haploinsufficiency on brain injury was evaluated at 4 weeks after HI, and the total tissue loss volume was reduced by 31.3% in Het mice (30.62±3.25 mm^3^, *n*=21) compared with Wt mice (44.60±5.13 mm^3^, *n*=10) (*P*=0.0244) (Figure 1c). These results indicate that the ubiquitous neuroprotective effect of CHCHD4 haploinsufficiency is long-lasting.

### CHCHD4 haploinsufficiency reduces neuronal cell death after HI

**Figure 2:**
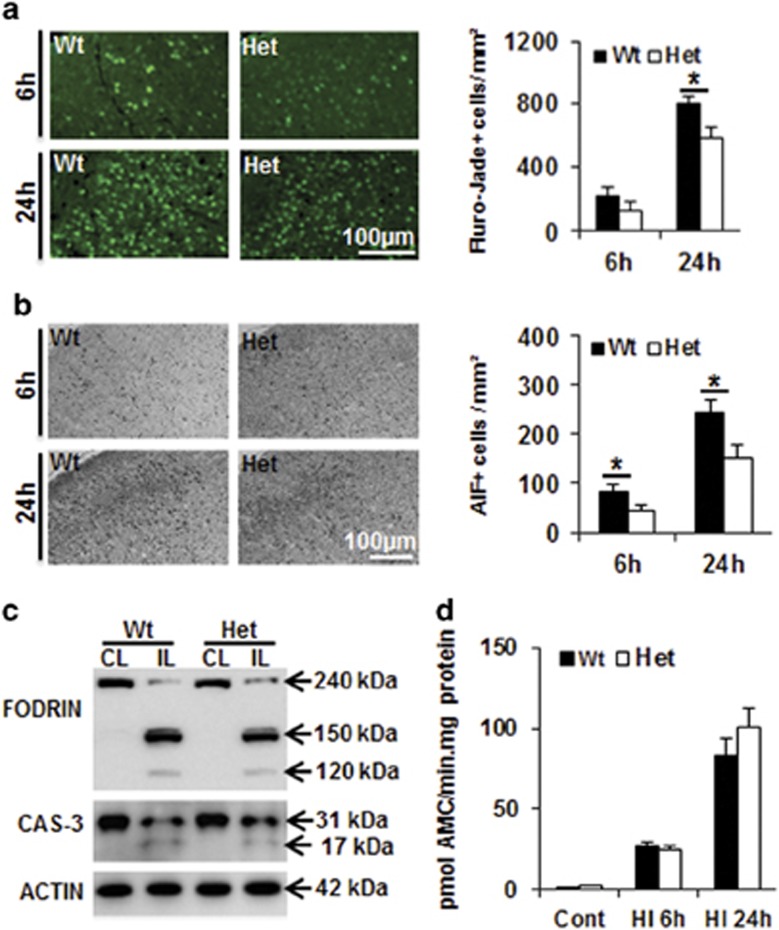
Effect of CHCHD4 reduction on cell death at 6 and 24 h after HI. (**a**) Representative Fluoro-Jade staining in the cortex (left) at 6 and 24 h after HI. The bar graph (right) shows the quantification of the number of Fluoro-Jade-positive dying cells. (**b**) Representative photomicrographs of AIF immunostaining in the cortex (left) at 6 and 24 h after HI. The bar graph (right) shows the number of AIF-positive cells (nuclear AIF immunostaining). (**c**) Representative western blotting of fodrin (top panel) and caspase-3 (middle panel) in both the ipsilateral (IL) and contralateral (CL) hemispheres at 24 h after HI in Wt and Het brains. Caspase-3 activation in the ipsilateral hemisphere is reflected by the appearance of a 17 kDa cleavage product in addition to the full-length 31 kDa protein (middle panel). Actin (bottom panel) was used as the loading control. Quantification of the cleaved bands did not reveal any significant difference between Wt and Het mice. (**d**) The bar graph shows caspase-3-like (DEVDase) activity in normal controls as well as ipsilateral hemispheres at 6 and 24 h after HI. There was no difference between Wt and Het brains in control or HI brains. *P<0.05

Neuronal cell death in the cortex was investigated by Fluoro-Jade staining, a non-specific neuronal marker of cell death, at 6 and 24 h after HI (Figure 2a). Fluoro-Jade-positive neurons, which were absent in the normal controls, became detectable at 6 h after HI. There was a tendency for more Fluoro-Jade-positive cells in Wt mice (*n*=7) compared to Het mice (*n*=5), but the difference was not statistically significant (*P*=0.1274). At 24 h after HI, the number of Fluoro-Jade-positive dying neurons increased dramatically, and the number of positive cells was significantly higher in Wt (810.8±38.9 cells per mm^2^, *n*=6) compared to Het mice (586.7±63.6 cells per mm^2^, *n*=7) (*P*=0.015) (Figure 2a). We further measured caspase-independent apoptotic cell death as indicated by AIF nuclear translocation.^23^ Compared to the Wt mice, the number of AIF-positive nuclei was reduced in the cortex of Het mice at both 6 and 24 h after HI (Figure 2b). Caspase-3 activation is a hallmark of caspase-dependent apoptotic cell death, while calpain activation mainly reflects necrotic cell death, and both of them induce fodrin degradation.^34^ In this study, immunoblots of cortex homogenates revealed fodrin degradation in the ipsilateral hemisphere at 24 h after HI, and there was no significant difference in the number of the fodrin breakdown products generated by calpain activation (145 and 150 kDa) or caspase-3 activation (120 kDa) (Figure 2c). Furthermore, caspase-3 cleavage (Figure 2b) and activity (Figure 2d) in the homogenates of the ipsilateral hemispheres were similar in Wt and Het mice at both 6 and 24 h after HI.

### CHCHD4 haploinsufficiency reduces proapoptotic protein release from mitochondria

**Figure 3:**
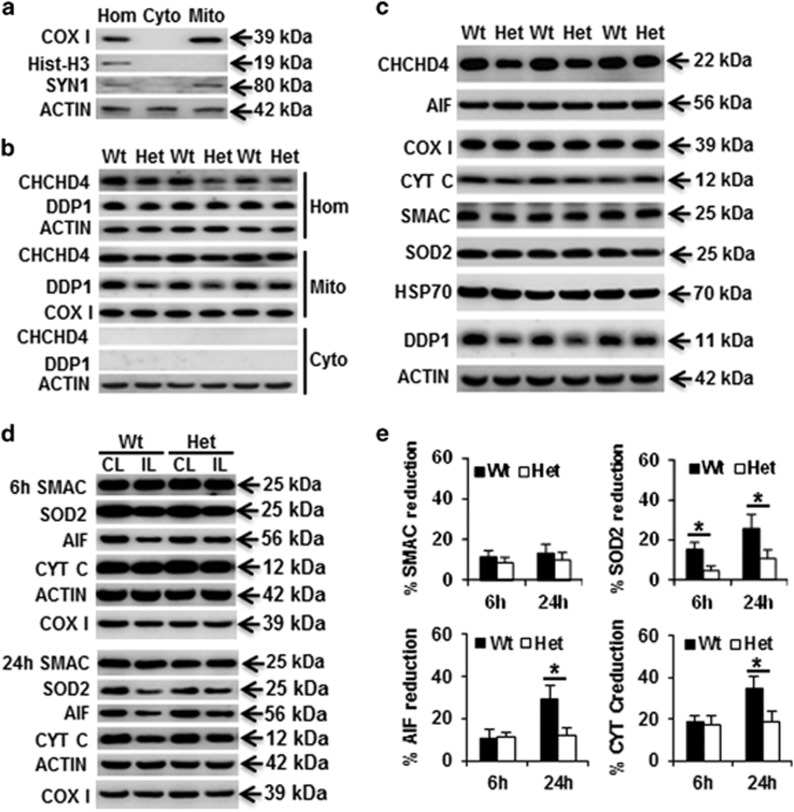
Expression of mitochondrial intermembrane space proteins. (**a**) Representative immunoblotting in brain homogenates (Hom), mitochondrial (Mito), and cytosolic (Cyto) fractions and the purity of mitochondrial fraction. (**b**) CHCHD4 immunoblotting in the mitochondrial and cytosolic fractions of brain homogenates. CHCHD4 protein was reduced by 25% in the brain homogenates of Het mice. (**c**) Immunoblotting of selected mitochondrial proteins from the mitochondrial fraction in P9 control mice. (**d**) Representative immunoblotting of Smac, SOD2, AIF, and Cyt *c* at 6 and 24 h after HI in the mitochondrial fraction of the ipsilateral (IL) and contralateral (CL) hemispheres. Actin was used as the loading control. (**e**) The bar graphs show the percentage of protein reduction in the ipsilateral hemisphere relative to the contralateral hemisphere after HI. *P<0.05

CHCHD4 is located in the mitochondrial intermembrane space and acts as a receptor to mediate the import of small proteins synthesized in the cytosol into the intermembrane space of the mitochondria. In the crude cytosolic fraction, there was very mild synaptosome contamination as indicated by synapsin1 (syn1) immunoblotting, but there was no nuclear contamination as indicated by histone H3 immunoblotting (Figure 3a). In postnatal day 9 (P9) Het mice, the total amount of CHCHD4 protein was about 25% less in the brain homogenate than the Wt littermates (Figure 3b). We next checked the expression of the mitochondrial proapoptotic proteins AIF, Cyt *c*, and second mitochondria-derived activator of caspases (Smac), as well as the mitochondrial oxidative stress-related proteins superoxide dismutase 2 (SOD2) and heat-shock protein 70 (Hsp70), none of which were significantly different in the Het mice compared with Wt littermates under physiological conditions (Figure 3c). The levels of all of these proteins decreased in the mitochondria in the ipsilateral hemisphere compared to the contralateral hemisphere after HI (Figure 3d). CHCHD4 haploinsufficiency prevented HI-induced AIF, Cyt *c*, and SOD2 loss in mitochondrial fraction after HI, but there was no obvious influence on Smac release from the mitochondria (Figure 3e).

### Impact of CHCHD4 haploinsufficiency on mitochondrial functions

**Figure 4:**
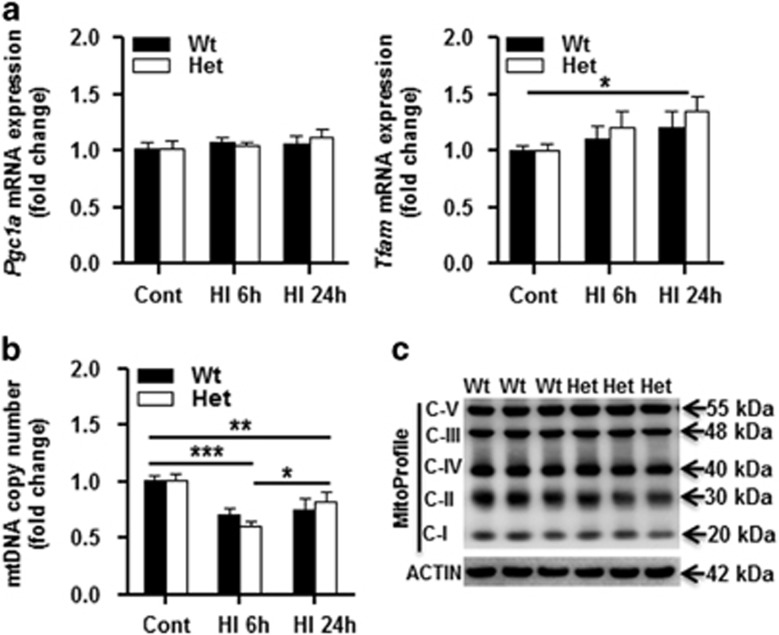
Mitochondrial biogenesis. (**a**) mRNA expression of mitochondrial biogenesis-related genes (*Pgc1α* and *Tfam*) normalized to the stable reference gene *Ywhaz* in Wt and Het mice at 6 and 24 h after HI. (**b**) mtDNA copy number decreased significantly at 6 h after HI compared to the controls. *n*=6 per group. (**c**) Immunoblotting of individual respiratory chain complexes CI, II, III, IV, and V subunits in the mitochondrial fraction of Wt and Het normal controls, *n*=6 per group. *P<0.05, **P<0.01, ***P<0.001

Mitochondrial biogenesis was checked by measuring the mRNA expression of peroxisome proliferator-activated receptor *γ* coactivator-1*α* (*Pgc1α*) and mitochondrial transcription factor A (*Tfam*), and we found no difference between Wt and Het mouse pups under physiological conditions or after HI (Figure 4a), and this agreed with the results of a mitochondrial DNA (mtDNA) copy number assay (Figure 4b) and with measurement of individual mitochondrial respiratory chain complex (CI, CII, CIII, CIV, CV) subunit expression in the mitochondrial fraction (Figure 4c). HI insult induced downregulation of mtDNA copy number at 6 h after HI, but there was no significant difference between Wt and Het mouse pups (Figure 4b).

**Figure 5:**
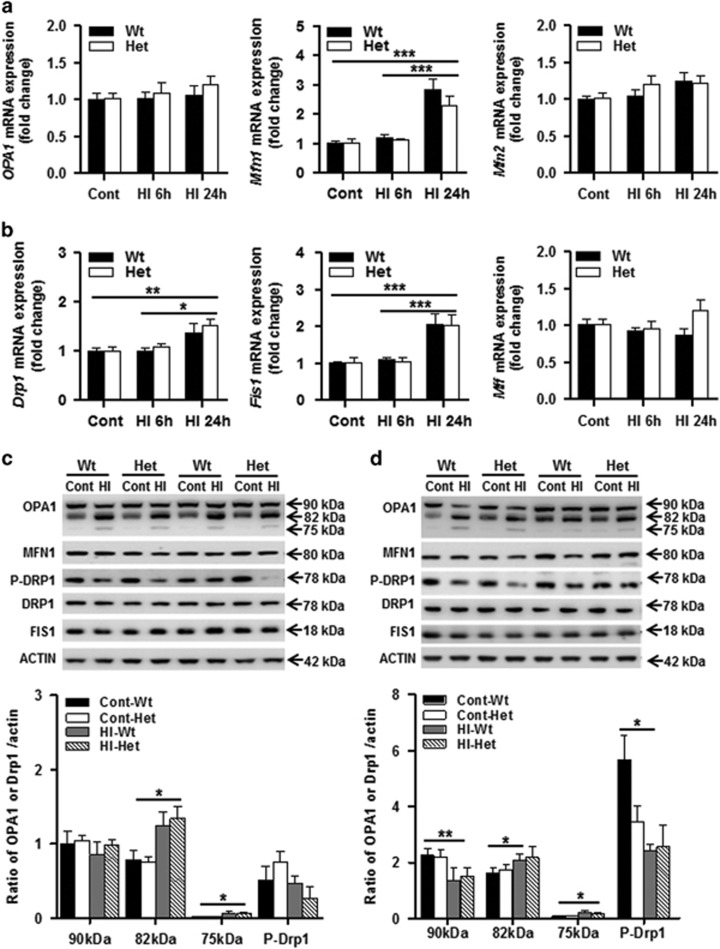
Mitochondrial fission and fusion. (**a**) The mRNA expression levels of the mitochondrial fusion genes (*Opa1*, *Mfn1*, and *Mfn2*) in Wt and Het mouse brains were quantified by RT-qPCR at 6 and 24 h after HI. (**b**) The mRNA expression levels of the mitochondrial fission genes (*Drp1*, *Fis1*, and *Mff*) in Wt and Het mouse brains were quantified by RT-qPCR at 6 and 24 h after HI. *n*=6 per group. (**c**) Immunoblotting of OPA1, MFN1, P-DRP1, and FIS1 in the homogenate of Wt and Het control (Cont), and at 6 h after HI. The 90 kDa upper band of OPA1 was not significantly changed at 6 h after HI, while the 82 kDa lower band and 75 kDa cleavage band increased significantly at 6 h after HI. The 82 kDa lower band increased significantly after HI compared with controls. The 75 kDa cleavage band increased significantly after HI. There was no significant change for MFN1, P-DRP1, or FIS1 between Wt and Het in either control or HI mice (*n*=6 per group). (**d**) Immunoblotting of OPA1, MFN1, P-DRP1, and FIS1 in the mitochondrial fraction of Wt and Het controls, and at 24 h after HI. The expression of 90 kDa OPA1 decreased significantly at 24 h after HI in both Wt and Het compared with the controls. The 75 kDa cleavage band of OPA1 increased significantly after HI, but there was no difference between Wt and Het in either controls or HI. P-DRP1 decreased after HI in the Wt mice compared with their controls, but no significant reduction in Het mice. There was no significant change for MFN1 or FIS1 (*n*=6 per group). *P<0.05, **P<0.01, ***P<0.001

Dynamic changes in mitochondria between Wt and Het mouse pups were examined by the mRNA expression of the mitochondrial optic atrophy 1 (*Opa1*) and mitofusion (*Mfn1, Mfn2*) fusion genes (Figure 5a), and the dynamin-1-like protein (*Drp1*), mitochondrial fission 1 (*Fis1*), and mitochondrial fission factor (*Mff*) fission genes (Figure 5b). There were no differences in gene expression under physiological conditions or at 6 h after HI between Wt and Het. *Mfn1* and *Fis1* gene expression increased dramatically at 24 h after HI, while no differences were seen for any of the other genes, and no differences were seen between Wt and Het mice. The protein expression of OPA1 was further checked, and the 82 kDa lower band increased significantly and the cleavage product of the 75 kDa band increased significantly at 6 h after HI (Figure 5c), while the 90 kDa upper band decreased significantly at 24 h after HI (Figure 5d). The increased cleavage product of the 75 kDa band indicates that mitochondrial fusion was reduced after HI, but there was no difference between Wt and Het under physiological conditions or after HI. The phosphor-DRP1(Ser637) (P-DRP1) protein expression decreased at 6 h and was more pronounced at 24 h after HI compared with controls, but no difference was seen between Wt and Het (Figures 5c and d). The decreased expression of P-DRP1 indicates that HI insult promoted mitochondrial fission.

**Figure 6:**
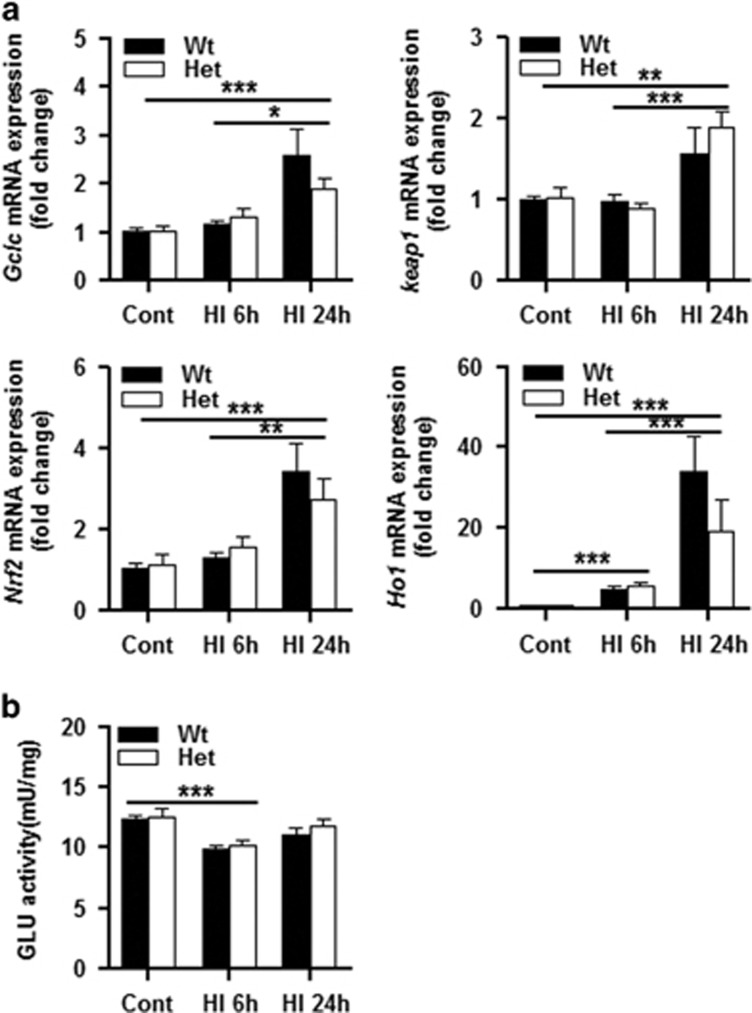
Transcription of Keap1–Nrf2 pathway-related genes. (**a**) *Nrf2*, *Keap1*, *Gclc*, and *Ho1* mRNA levels were quantified by qRT-PCR in P9 normal brain tissue as well as at 6 and 24 h after HI in Wt and Het mice (*n*=6 per group). No significant changes were observed between the genotypes. (**b**) Glutathione reductase (GLU) activity in the mitochondrial fraction was decreased at 6 h after HI compared with controls, and there was no significant difference between the genotypes under physiological conditions or after HI insult. *P<0.05, **P<0.01, ***P<0.001

To determine if CHCHD4 haploinsufficiency has an effect on cellular redox capacity, we used RT-qPCR to measure the change in Keap1-NRF2 pathway-related genes at the mRNA level. Under physiological conditions, the transcription of these genes did not change significantly in Het mice compared to Wt mice (Figure 6a). The activity of glutathione reductase was decreased at 6 h after HI, and there was no difference in glutathione reductase activity between Wt and Het mice under physiological conditions or after HI (Figure 6b). Thus, CHCHD4 haploinsufficiency to 25% apparently does not influence the mitochondrial redox capacity.

### CHCHD4 haploinsufficiency has no influence on the p53 pathway

**Figure 7:**
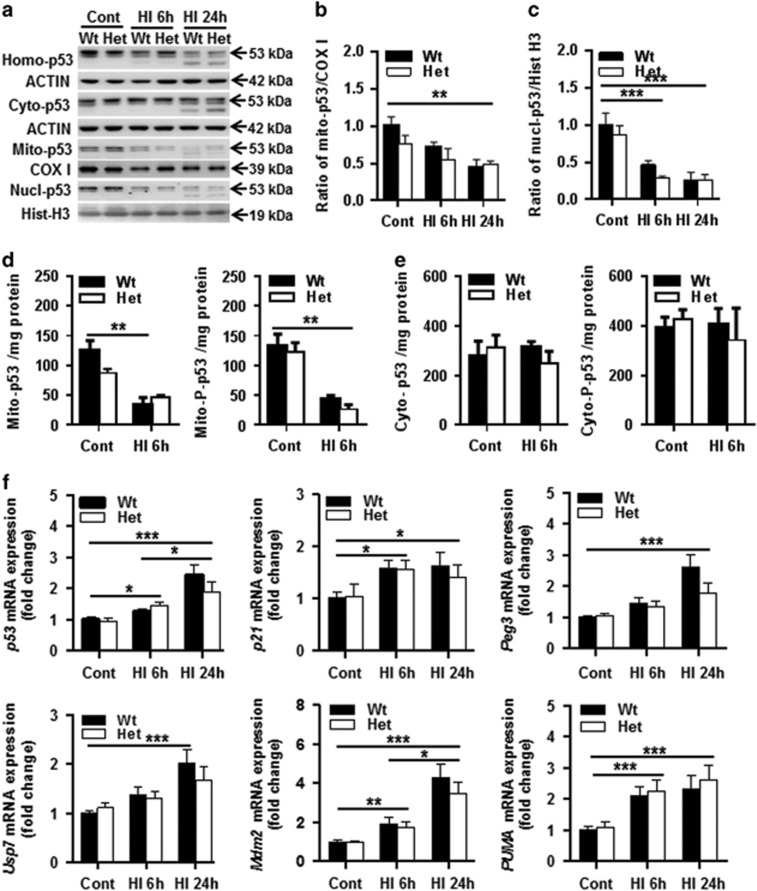
Effect of CHCHD4 reduction on p53 expression. (**a**) Immunoblotting of p53 in the homogenate (top panel, Homo-p53), cytosolic (Cyto-p53), mitochondrial (Mito-p53), and nuclear fraction (bottom panel, Nucl-p53) of normal control (Cont) mice and at 6 or 24 h after HI. The p53 band was decreased in both mitochondrial and nuclear fractions, and was more pronounced at 24 h after HI in both the Wt and Het groups. (**b**) Bar graphs show the quantification of p53 immunoblotting in the mitochondrial fraction. (**c**) Bar graphs show the quantification of p53 immunoblotting in the nuclear fraction of the Wt and Het groups (*n*=6 per group). (**d**) ELISA assay of total and phosphorylated p53 in the mitochondrial fraction of controls and at 6 h after HI in both Wt and Het mice. (**e**) Total and phosphorylated p53 in the cytosolic fraction of controls and at 6 h after HI in both Wt and Het mice (*n*=5 per group). (**f**) The mRNA expression levels of p53 pathway-related genes, including *p53*, *p21*, *Peg3*, *PUMA*, *Usp7*, and *Mdm2*, were quantified by RT-qPCR both in Wt and Het mice, and normalized with the reference gene *Ywhaz* at different time points after HI. *n*=6 for Wt control group, *n*=5 for Het control group, *n*=6 for both the Wt and Het groups at 6 h after HI, and *n*=6 for Wt and *n*=5 for Het at 24 h after HI. *P<0.05, **P<0.01, ***P<0.001

CHCHD4 has been shown to mediate p53 translocation into mitochondria,^35^ and p53 mediates neuronal cell death in the neonatal rat brain after HI.^36^ We checked p53 expression in the mitochondria, cytosol, and nuclear fraction under physiological conditions and after HI. There was no significant difference in p53 expression in different cellular fractions between Wt and Het mouse pups under physiological conditions (Figures 7a–e). After HI, p53 levels in the mitochondria (Figures 7b, d) and nuclear fractions (Figure 7c) decreased. There was no significant change in the cytosolic fractions except a cleavage band around 43 kDa at 24 h after HI (Figures 7a, e). However, there was no difference in the expression of total p53 and phosphorylated p53 (pS15) in the different cellular fractions after HI between Wt and Het mice (Figures 7a–e). These data indicate that mild CHCHD4 downregulation has no effect on p53 expression in different cellular fractions, neither under physiological condition nor after HI in young mice. We next checked the mRNA expression of p53 pathway-related genes (Figure 7f). The mRNA expression of the *p53*, *p21*, *PUMA* (p53-upregulated modulator of apoptosis), and *Mdm2* genes increased significantly at 6  and 24 h after HI compared with controls (*P*=0.012 and *P*<0.001 for *p53*, *P*=0.015 and *P*=0.042 for *p21*, *P*<0.001 and *P*<0.001 for *PUMA*, *P*=0.0024 and *P*<0.001 for *Mdm2*), and *p53* and *Mdm2* mRNA expression at 24 h was significantly higher than at 6 h after HI (*P*=0.013 and *P*=0.046, respectively). There was no significant difference between the Wt and Het groups for any of the selected genes either under physiological conditions or after HI. For *Peg3* and *Usp7* mRNA expression, significant differences were seen between controls and 24 h after HI in Wt mice (both *P*<0.001).

### CHCHD4 haploinsufficiency has no influence on neural stem cell proliferation or differentiation

**Figure 8:**
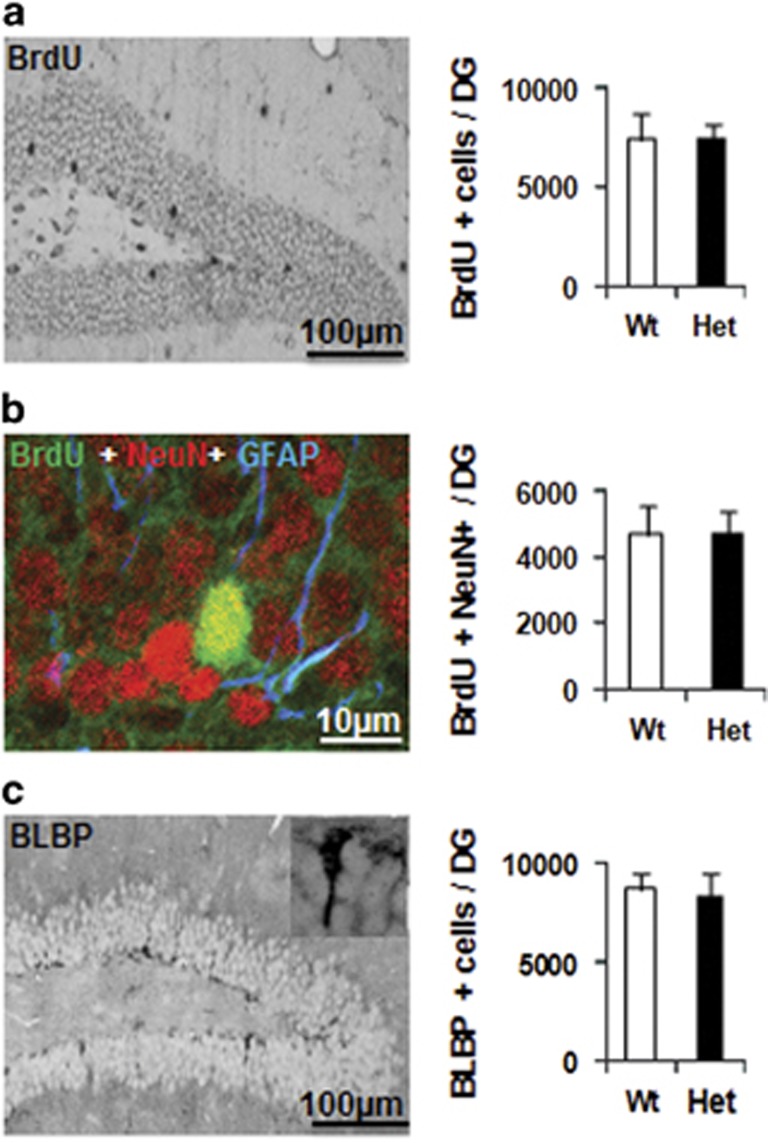
Effect of CHCHD4 reduction on cell proliferation and differentiation. (**a**) Representative photomicrographs of BrdU staining in the dentate gyrus (DG) at 4 weeks after BrdU injections. The bar graph shows the total number of BrdU-labeled cells. (**b**) Representative BrdU/NeuN/GFAP staining, and the bar graph shows newborn neurons (BrdU^+^/NeuN^+^). (**c**) Representative photomicrographs of BLBP staining in the DG at 4 weeks after BrdU injections. The bar graph shows the total number of BLBP-labeled cells in the subgranular zone

Neural stem cell proliferation and survival over the course of 4 weeks was measured by 5-bromo-2-deoxyuridine (BrdU) labeling, and quantification in the granular layer of the dentate gyrus did not show any differences between Wt and Het mice (Figure 8a). The neuronal differentiation of the surviving newborn cells was the same in Wt and Het control mice (Figure 8b). There were also no differences in the number of neural precursors between Wt and Het control mice as indicated by BLBP labeling in the subgranular zone (Figure 8c).

## Discussion

Neuronal cell death in the immature brain after HI includes apoptosis, necrosis, autophagic cell death, and various hybrid morphological features, and the type of cell death depends on the severity of the insult.^11, 37, 38^ Previous studies have shown that apoptosis plays a more prominent role under pathological conditions in the immature brain compared to the adult brain, and this includes both caspase-dependent and caspase-independent apoptotic pathways. Combined inhibition of both pathways provides synergistic protection against neonatal HI brain injury,^23^ and our previous study showed that caspase-independent apoptosis is positively correlated with brain injury after HI.^22^ This indicates that AIF plays an important role in the process of neuronal cell death and brain injury.^23, 24, 26, 39^ However, AIF, as a mitochondrial intermembrane space protein, plays a vital role in cell survival, proliferation, and mitochondrial integrity,^17^ and now it is established that this pro-survival function of AIF requires, at least, its physical and functional interaction with the CHCHD4-dependent import pathway.^21, 29^ AIF downregulation reduces CHCHD4 expression^21, 27^ and reduces the extent of HI brain injury.^23^ It is still unknown whether the neuroprotection is due to the reduced AIF release from the mitochondria or reduced CHCHD4 expression.^21^ In the current study, CHCHD4 was downregulated by 25% in *CHCHD4*^*+/−*^ mouse pups without influencing AIF protein expression, and this downregulation reduced the extent of HI brain injury by 21%, and the protective effect was even more pronounced with longer recovery when the injury was evaluated at 4 weeks after HI. This indicates that CHCHD4 haploinsufficiency influences mitochondrial function, mitochondrial membrane potential, or other non-mitochondrial signaling pathways.^28, 29^

Neuronal cell death, as indicated by Fluoro-Jade labeling, was decreased significantly at 24 h after HI in Het mouse pups, and this was correlated with reduced AIF-positive nuclear staining, but caspase-3 activation was not significantly different between Het and Wt mouse pups. These results indicate that CHCHD4 haploinsufficiency reduces AIF release from the mitochondria and subsequent nuclear translocation, and this was confirmed by AIF immunoblotting. The reduction of other mitochondrial proteins after HI, such as Cyt *c*, was attenuated by CHCHD4 haploinsufficiency, but no significant effect was seen for Smac. This could be related to specific functional interactions between CHCHD4 and the target mitochondrial intermembrane proteins. However, reduced AIF and Cyt *c* release from the mitochondria can partly explain the neuroprotection afforded by CHCHD4 downregulation. SOD2, which is one of the most important oxidative stress defense enzymes and catalyzes the dismutation of superoxide radicals into hydrogen peroxide and oxygen, is exclusively located in the mitochondrial matrix. It has been shown that both mRNA and protein expression of SOD2 rapidly decreases during the early reperfusion period after cerebral ischemic insult, and this was attributed to deactivation of signal transducer and activator of transcription.^40^ In this study, CHCHD4 downregulation prevented HI-induced SOD2 reduction, which might affect Cyt *c* translocation and downstream caspase activation in the mitochondrial-dependent cell death pathway.^41^ However, the detailed mechanism underlying the regulation of SOD2 expression after cerebral HI in the immature brain is not fully understood.

Mitochondria have multiple functions throughout brain development, especially the regulation of ATP production and cell death, and mitochondrial fusion, fission, and biogenesis are crucial for functional recovery after injury.^7^ Mitochondrial biogenesis contributes to energy homeostasis by generating ATP through a series of oxidative phosphorylation reactions. Oxidative phosphorylation comprises four respiratory chain complexes, two mobile electron carriers, and ATP synthase and converts inorganic phosphate and ADP into ATP. CHCHD4 catalyzes the oxidative folding of disulfide-containing proteins in the mitochondria, which are required for the biogenesis of respiratory chain complexes.^21, 29^ In the current study, mitochondrial respiration showed no significant defects in the *CHCHD4*^*+/−*^ Het pups. There was no significant change in mitochondrial biogenesis as indicated by mtDNA content and protein expression of respiratory chain complexes, and by the expression of mitochondrial biogenesis-related genes in the neonatal brain, which is different from a previous report that a >50% deficit of CHCHD4 reduced the biogenesis of respiratory chain complexes in human U2OS cancer cells and mouse embryonic stem cells.^21^ The difference in mitochondrial biogenesis in mice of different ages is probably related to the degree of CHCHD4 reduction in specific organs and the possibility of compensation by the remaining CHCHD4 proteins.

The dynamic changes between mitochondrial fusion and fission are important for mitochondrial function, response to stress condition and cell survival.^42^ In the pathological condition of HI, maintaining mitochondrial fusion might have a protective effect.^43, 44^ In the current study, the expression of the mitochondrial fusion genes *Opa1*, *Mfn1*, and *Mfn2* was not different between Wt and Het mice, and was not different between physiological and pathological conditions. Furthermore, the expression of the mitochondrial fission-related genes *Drp1*, *Fis1*, and *Mff* and phosphorylation of DRP1 was not different between Wt and Het mouse pups. These results suggest that the neuroprotection afforded by CHCHD4 haploinsufficiency cannot be explained by a change in mitochondrial fusion or fission after HI.

The p53 protein regulates diverse cellular processes, including cell death, redox homeostasis, and metabolism, under both physiological and pathological conditions.^45, 46^ Studies have shown that p53 is involved in neonatal rat cerebral HI brain injury and that p53 translocates and accumulates in the mitochondria at 3 or 6 h after HI.^36, 47^ Furthermore, p53 inhibitors block mitochondrial accumulation of p53 and reduce mitochondrial membrane pore opening as well as the release of proapoptotic proteins such as Cyt *c* from mitochondria that lead to neuronal cell death and brain injury.^36^ In this mouse study, we did not find p53 to be translocated into the mitochondria after HI, which is in contrast with previous reports in rats after cerebral ischemia.^36, 47, 48^ Furthermore, we did not find any accumulation of p53 in the nuclear fraction;^36^ instead, we actually found a reduction of p53 in the nuclear fraction of neonatal mice after HI. We did not find significant changes of p53 in the cytosolic fraction after HI, which is different from previous reports that showed an increase in cytosolic p53 in neonatal rats after HI^47^ and a decrease in adult rats after cerebral ischemia.^48^ The discrepancy between these reports suggests that p53 translocation between the cellular compartments is age, species, and injury dependent and might be related to different cell death mechanisms.

It has been shown that the translocation of p53 into the mitochondria is mediated by CHCHD4, and decreased CHCHD4 expression prevents this mitochondrial translocation while augmenting the nuclear localization and activity of p53.^35^ In this study, we speculated that reduced neonatal HI brain injury after CHCHD4 downregulation might be related to reduced translocation of p53 into the mitochondria. However, in the Het mouse pups with a 25% reduction in CHCHD4 expression, there was no significant influence on p53 protein expression or cellular distribution under physiological conditions or after HI, which might be because of the mild CHCHD4 downregulation—and thus because the reduced CHCHD4 function can be compensated for—or this might indicate that the p53 pathway does not play a crucial role in cell death and brain injury in neonatal mice after HI.

In conclusion, our results demonstrate that CHCHD4 downregulation reduces neuronal cell death and caspase-independent apoptotic cell death, which affords long-term neuroprotection. Thus, the CHCHD4 import pathway is being revealed as a therapeutic target for preventing and treating neonatal HI brain injury, and for this reason, future investigations will be necessary for the characterization of the metabolic basis of the neuroprotection provided by the downregulation of CHCHD4 and for the identification of the specific set of CHCHD4 substrates that could play key roles in the observed phenomenon.^29^

## Materials and Methods

### Induction of HI brain injury

P9, Wt, or Het CHCHD4 knockout littermates^21^ of both sexes were anesthetized with isoflurane (5% for induction, 1.5–3.0% for maintenance) in a mixture of nitrous oxide and oxygen (1 : 1), and the duration of anesthesia was <5 min. The left common carotid artery was cut between double ligatures of prolene sutures (6.0), and the wounds were infiltrated with lidocaine for analgesia after the surgical procedures. The pups were returned to their dams for 60 min and then placed in a chamber perfused with a humidified gas mixture (10% oxygen in nitrogen) for 50 min at 36 °C. Following hypoxic exposure, the pups were returned to their dams. Control pups were not subjected to ligation or hypoxia and were selected randomly from the litters. The pups with bleeding during the operation were excluded from the study. Because of the inherent variation of injury severity in the model, the two groups needed roughly 20 animals each for injury evaluation. The investigators were blinded to the groups because the genotyping was done when the pups were killed. All animal experiments were approved by the Animal Ethics Committee of Gothenburg (90–2011), and all the experiments were in accordance with the guidelines for laboratory experiments of the university.

### Genotyping

Genomic DNA was isolated from tail samples using DNA extraction kits (DNeasy, Cat. No. 69506, Qiagen, Hilden, Germany) according to the instructions of the manufacturer. The DNA concentration was measured by using a Nanodrop (Thermo Scientific, Waltham, MA, USA) and adjusted to around 50 ng/*μ*l. The reaction mixture for genotyping contained 1 *μ*l of genomic DNA, 0.2 mM dNTP, 4 *μ*l 5 × green PCR buffer (250 mM Tris-HCl, pH 8.3, 375 mM KCl, 15 mM MgCl_2_; Promega), 1 U of Taq DNA Polymerase (Promega, Madison, WL, USA), and either 0.5 *μ*M of common and Wt primers or 0.5 *μ*M of common and mutant primers. The PCR cycles were 95 °C for 30 s, 61°C for 30 s, and 72 °C for 45 s for 40 cycles. The following primers were used: IST11943B12-r common: 5′-GTGCTCCTCATAGGGATCATTGG-3′, Wt: IST11943B12-f 5′-TGGGCTGGTTAGTCAGTGATTGG-3′, and mutant: 5′-AAATGGCGTTACTTAAGCTAGCTTGC-3′. PCR products were separated on a 1.5% agarose gel containing SYBR green (1:10 000 dilution). A 100 basepair (bp) ladder was used to verify the sizes of the PCR products. The gels were imaged with a LAS 3000 cooled CCD camera (Fujifilm, Tokyo, Japan). CHCHD4-mutant mice were identified by the presence of a single 205 bp DNA band, and Wt mice were identified by a single 213 bp DNA band.

### BrdU administration

The thymidine analog BrdU (Roche, Mannheim, Germany, 5 mg/ml dissolved in 0.9% saline) was prepared fresh prior to use and injected intraperitoneally (50 mg/kg) on P10 and P11. The mice were killed on P38 to evaluate the survival of newly proliferated cells.

### Injury evaluation

Brain injury was evaluated by measuring the volume of total hemispheric tissue loss and the pathological scoring in different brain regions stained for microtubule-associated protein 2 (MAP2). Injury evaluation was done by a person who was blinded to the genotype and group. The MAP2-positive and MAP2-negative areas in each section were measured using Micro Image (Olympus). The tissue volume was calculated from the MAP2-positive areas according to the Cavalieri principle using the following formula: *V*=Σ*A* × *P* × *T*, where *V*=the total volume, Σ*A*=the sum of area measurement, *P*=the inverse of the sampling fraction, and *T*=the section thickness. The total hemispheric tissue loss was calculated as the MAP2-positive volume in the contralateral hemisphere minus the MAP2-positive volume in the ipsilateral hemisphere.

Brain injury at 72 h post HI in different regions was evaluated using a semi-quantitative neuropathological scoring system.^37^ Briefly, sections were stained for MAP2 and scored by an observer blinded to the genotyping of the animals. The cortical injury was graded from 0 to 4 with 0 being no observable injury and 4 being confluent infarction encompassing most of the cerebral cortex. The damage in the hippocampus, striatum, and thalamus was assessed both with respect to hypotrophy (shrinkage) (possible score 0–3) and observable cell injury/infarction (possible score 0–3) resulting in a neuropathological score for each brain region (possible score 0–6). The total score (0–22) was the sum of the scores for all four regions.

### Sample preparation for immunoblotting and enzyme activity analysis

Animals were killed by decapitation at 6 or 24 h after HI. Control animals were killed on P9. The brains were rapidly dissected out on a bed of ice, and the parietal cortex (including the hippocampus) was dissected out from each hemisphere and ice-cold isolation buffer was added (15 mM Tris-HCl, pH 7.6, 320 mM sucrose, 1 mM dithiothreitol, 1 mM MgCl_2,_ 3 mM EDTA-K, 0.5% protease inhibitor cocktail (P8340; Sigma, Stockholm, Sweden), and 2.5 *μ*M cyclosporin A). Homogenization was performed gently by hand in a 2 ml glass/glass homogenizer (Merck Eurolab, Dorset, UK) with two different pestles with a total clearance of 0.12  and 0.05 mm, respectively (10 strokes each). The homogenates were centrifuged at 800 × *g* for 10 min at 4 °C. The pellets were washed in homogenizing buffer and recentrifuged at 800 × *g* for 15 min at 4 °C, producing a crude nuclear pellet. The supernatant from the first centrifugation was further centrifuged at 9200 × *g* for 15 min at 4 °C, producing a crude cytosolic fraction in the supernatant, and a mitochondrial and synaptosomal fraction in the pellet, which was washed in homogenizing buffer and recentrifuged at 9200 × *g* for 15 min at 4 °C.^49^ All fractions were stored at −80 °C.

### Caspase activity assay

The protein concentrations were determined with the BCA protein assay adapted for microplates. Homogenate samples (40 *μ*l) were mixed with 60 *μ*l of extraction buffer as described earlier. Cleavage of Ac-DEVD-AMC (for caspase-3-like activity, Peptide Institute, Osaka, Japan) was measured with an excitation wavelength of 380 nm and an emission wavelength of 460 nm, and expressed as pmol AMC released per mg protein and minute.^50^

### Glutathione reductase activity assay

The samples of the enriched mitochondrial fraction were used for glutathione reductase activity measurement according to the manufacturer’s instructions (K761-200, Biovision, Milpitas, CA, USA). Briefly, 100 *μ*l of enriched mitochondrial fraction was mixed with 5 *μ*l 3% H_2_O_2_ and incubated at 25 °C for 5 min, and then 5 *μ*l of catalase was added and the solution was mixed and incubated at 25 °C for 5 min. A total volume of 25 *μ*l of the pretreated samples was placed into a 96-well plate, and assay buffer was added to 50 *μ*l. A total of 50 *μ*l reaction mix was added, and the absorbance was measured immediately at 450 nm in kinetic mode for 60 min at 37 °C. The activity was calculated according to the standard curve and expressed as mU/mg protein.

### Phosphorylated and total p53 assay

Phosphorylated p53 (pS15) and total p53 protein were semi-quantitatively measured in mitochondrial and crude cytosolic fractions from both controls and 6 h after HI of Wt and Het mouse samples. The p53 protein assay was performed as per manufacturer’s recommendations (p53 (pS15)+total p53 SimpleStep ELISA Kit, Abcam, ab205713, Cambridge, UK). The serially diluted control and diluted samples, both 50 *μ*l, were mixed with 25 *μ*l capture antibody and 25 *μ*l detector antibody, and then incubated for 1 h at room temperature on a plate shaker set to 400 rpm. After three washes, 100 *μ*l TMB (3,3',5,5'-tetramethylbenzidine) substrate was added to each well. Samples were incubated for 15 min in the dark on a plate shaker at 400 rpm, and the reactions were stopped by adding 100 *μ*l stop solution to each well. After shaking for 1 min, the OD was recorded at 450 nm. Data were normalized by protein concentration and expressed as units/mg protein.

### Immunoblotting

A total of 65 *μ*l of each brain cellular fraction sample was mixed with 25 *μ*l NuPAGE LDS 4 × sample buffer and 10 *μ*l reducing agent, and then heated at 70 °C for 10 min. Individual samples were run on 4–12% NuPAGE Bis-Tris gels (Novex, San Diego, CA, USA) and transferred to reinforced nitrocellulose membranes (Schleicher & Schuell, Dassel, Germany). After blocking with 30 mM Tris-HCl (pH 7.5), 100 mM NaCl, and 0.1% Tween 20 (TBS-T) containing 5% fat-free milk powder for 1 h at room temperature, the membranes were incubated with primary antibodies: anti-AIF (sc-9416, 1:1000 dilution, 0.2 *μ*g/ml, goat polyclonal antibody, Santa Cruz Biotechnology, Dallas, TX, USA), anti-caspase-3 (H-277, 1:1000, rabbit polyclonal antibody, Santa Cruz Biotechnology), anti-Cyt *c* (clone 7H8.2C12, 1:500, Pharmingen, San Diego, CA, USA), anti-actin 200, rabbit polyclonal antibody, Sigma), anti-superoxide dismutase II (Mn-SOD/SOD2, clone 2A1, 1:1000, 0.5 *μ*g/ml, Lab Frontier, Seoul, Korea), anti-fodrin (clone AA6, 1:500, 0.2 *μ*g/ml, BIOMOL, Plymouth Meeting, PA, USA), anti-OxPhos Complex I 39 kDa subunit (clone 20C11, 1:1000, 0.5 *μ*g/ml, Molecular Probes, Eugene, OR, USA), total oxidative phosphorylation system rodent western blot antibody cocktail (MS604, 1:250, MitoSciences, Eugene, OR, USA), anti-Smac (sc-227766, 1:500, rabbit polyclonal antibody, Santa Cruz Biotechnology), anti-Hsp70 (sc-7298, 1:500, mouse monoclonal antibody, Santa Cruz Biotechnology), anti-p53 (sc-99, 1:500, mouse monoclonal antibody, Santa Cruz Biotechnology), anti-histone H3 (06-755, 1:500, rabbit polyclonal antibody, Millipore, Temecula, CA, USA), anti-phospho-DRP1(Ser637) (4867, 1:1000, rabbit polyclonal antibody, Cell Signaling, Danvers, MA, USA); anti-DRP1(C-5) (sc-271583, 1:300, mouse monoclonal antibody, Santa Cruz Biotechnology); anti-syn1(N-19) (sc-7379, 1:500, goat polyclonal antibody, Santa Cruz Biotechnology); (anti-OPA1 (612606, 1:1000, mouse monoclonal antibody, BD Bioscience, San Jose, CA, USA), anti-MFN1 (NBP1-71775, 1:500, mouse monoclonal antibody, Novusbio, Littleton, CO, USA), anti-FIS1 (FL-152) (sc-98900, 1:500, rabbit polyclonal antibody, Santa Cruz Biotechnology), or anti-TIMM8A (DDP1, 11179-1-AP, 1:500, rabbit polyclonal antibody, Proteintech, Rosemont, IL, USA) at room temperature for 60 min. After washing, the membranes were incubated with a peroxidase-labeled secondary antibody for 30 min at room temperature (goat anti-rabbit 1:2000, horse anti-goat 1:2000, or horse anti-mouse 1:4000). Immunoreactive species were visualized using the Super Signal West Dura substrate (Pierce, Rockford, IL, USA) and a LAS 3000 cooled CCD camera (Fujifilm).

### Tissue preparation and immunohistochemistry staining

Animals were deeply anesthetized with 50 mg/ml phenobarbital and killed at 6, 24, 72 h, or 4 weeks after HI. Control animals were killed on P9, P12, or P38. The brains were perfusion-fixed with a 5% formaldehyde solution buffered with sodium phosphate at pH 7.4 and stabilized with methanol (Histofix, Histolab Products AB, Västra Frölunda, Sweden) through the ascending aorta for 5 min. The brains were rapidly removed and immersion-fixed at 4 °C for 24 h. The brains were dehydrated with xylene and graded ethanol, paraffin-embedded, and serial-cut into 5 *μ*m frontal sections. Antigen retrieval was performed by heating the sections in 10 mM boiling sodium citrate buffer (pH 6.0) for 10 min. Non-specific binding was blocked for 30 min with 4% goat or horse serum (depending on the species used to raise the secondary antibody) in PBS. Monoclonal rat anti-BrdU (clone: BU1/75, 1:100, 5 *μ*g/ml; Oxford Biotechnology Ltd, Oxfordshire, UK), monoclonal mouse anti-MAP-2 (clone HM-2, 1:1000, Sigma, Saint Louis, MO, USA), goat anti-AIF (sc-9416, 1:100, Santa Cruz Biotechnology), or rabbit anti-BLBP (ABN14, 1:600, Millipore) primary antibody was applied and incubated at 20 °C for 60 min, followed by the appropriate biotinylated donkey anti-rat IgG (H+L) (1:200, 5.5 *μ*g/ml; Jackson ImmunoResearch Lab, West Grove, PA, USA), horse anti-mouse (1:200, Vector Laboratories, Burlingame, CA, USA), goat anti-rabbit (1:150, for CHCHD4 and BLBP), or horse anti-goat (1:200, for AIF) secondary antibody for 60 min at 20 °C. Endogenous peroxidase activity was blocked with 3% H_2_O_2_ in PBS for 10 min. Visualization was performed using Vectastain ABC Elite (Vector Laboratories) with 0.5 mg/ml 3,3’-diaminobenzidine enhanced with 15 mg/ml ammonium nickel sulfate, 2 mg/ml beta-d glucose, 0.4 mg/ml ammonium chloride, and 0.01 mg/ml beta-glucose oxidase (all from Sigma).

The phenotype of BrdU-labeled cells was determined using antibodies against NeuN and S100*ß* to detect mature neurons and astrocytes, respectively. Antigen recovery was performed as above followed by incubation with rat anti-BrdU (clone: BU1/75, 1:100, 5 *μ*g/ml; Oxford Biotechnology Ltd) together with mouse anti-NeuN (clone: MAB377, 1:200, 5 *μ*g/ml; Chemicon, Temecula, CA, USA) and rabbit anti-S-100*ß* (1:1000; Swant, Bellinzona, Switzerland) in PBS at 20 °C for 60 min. After washing, the sections were incubated with the secondary antibodies Alexa Fluor 488 donkey anti-rat IgG (H+L), combined with Alexa 555 donkey anti-mouse IgG (H+L), and Alexa 647 donkey anti-rabbit IgG (H+L) at 20 °C for 60 min. All secondary antibodies were from Jackson ImmunoResearch Lab and were diluted 1:500. After washing, the sections were mounted using Vectashield mounting medium.

### Fluoro-Jade staining

Brain sections were incubated sequentially in each of the following solutions for the times indicated: 100% alcohol, 3 min; 70% alcohol, 1 min; distilled water, 1 min; 0.06% potassium permanganate, 15 min; distilled water, 1 min; 0.001% Fluoro-Jade in 0.09% acetic acid, 30 min; and distilled water, 2 × 1 min. Sections were cover slipped and kept in the dark.

### Mitochondrial biogenesis, fission, and fusion and NRF2 and p53 pathway-related gene expression

Total RNA was isolated with an RNA isolation kit (RNeasy Mini Kit, Qiagen, 74104) according to the manufacturer’s instructions. The concentration and purity as well as the integrity of the RNA were determined as described previously.^12^ One microgram of total RNA was reverse transcribed using the QuantiTect Reverse Transcription kit (Qiagen, 205311). Quantitative real-time PCR was performed using the LightCycler 480 instrument (Roche Diagnostics, Mannheim, Germany) and the SYBR Green technique according to the manufacturer’s instructions. The primers used in the qRT-PCR reactions were designed by Beacon Designer software (free trial, PREMIER Biosoft, Palo Alto, CA, USA) and included the mitochondrial biogenesis genes *Pgc1α* (sense: 5′-CCAGGTCAAGATCAAGGT-3′, antisense: 5′-CGTGCTCATAGGCTTCATA-3′), *Tfam* (sense: 5′-ACCTCGTTCAGCATATAACATT-3′, antisense: 5′-ATCACTTCGTCCAACTTCAG-3′), and *Nrf1* (sense: 5′-CCACAGGAGGTTAATTCAGAG-3′, antisense: 5′-ACATCACTGCGGACATTG-3′); the mitochondrial fission and fusion genes *Drp1* (sense: 5′-TGCTCAGTATCAGTCTCTTC-3′, antisense: 5′-GGTTCCTTCAATCGTGTTAC-3′), *Fis1* (sense: 5′-ATGAAGAAAGATGGACTGGTAG-3′, antisense: 5′-GGATTTGGACTTGGAGACA-3′), *Mff* (sense: 5′-ATTCAATCACTGTAGCGTTCT-3′, antisense: 5′-CTTTATATTTCCAGGTGTTGAGAC-3′), *Opa1* (sense: 5′-CCTGTGAAGTCTGCCAAT-3′, antisense: 5′-TTAGAGAAGAGAACTGCTGAAAT-3′), *Mfn1* (sense: 5′-CACTGATGAACACGGAGAA-3′, antisense: 5′-CGACGGACTTACAACCTT-3′), and *Mfn2* (sense: 5′-CGCCATATAGAGGAAGGT-3′, antisense: 5′-CGCATAGATACAGGAAGAAG-3′); the Keap-Nrf2 pathway-related genes *Gclc* (sense: 5′-TAGAACACGGGAGGAGAG-3′, antisense: 5′-CCACACTTAGACAGGTAGC-3′), *Keap1* (sense: 5′-ATGGGAATAAAGAATGGAGTAGG-3′, antisense: 5′-ATGGCAAGCAGAGACAATAG-3′), *Nrf2* (sense: 5′-GTGCTCCTATGCGTGAAT-3′, antisense: 5′-CGGCTTGAATGTTTGTCTTT-3′), and *Ho1* (sense: 5′-TACACATCCAAGCCGAGAA-3′, antisense: 5′-TACAAGGAAGCCATCACCA-3′); and the p53 pathway-related genes *p53* (sense: 5′-ACAAGAAGTCACAGCACAT-3′, antisense: 5′-CCAGATACTCGGGATACAAAT-3′), *p21* (sense: 5′-AAGTGTGCCGTTGTCTCT-3′, antisense: 5′-AAGTCAAAGTTCCACCGTT-3′), *Peg3* (sense: 5′-AAGGAAGAGGCGTTACCA-3′, antisense: 5′-TCATCTCAGCACCACACT-3′), *Usp7* (sense: 5′-TGAGTGAGTCGGTCCTTAG-3′, antisense: 5′-TGGAGTCAGATTCAGCATTG-3′), *Mdm2* (sense: 5′-TGGCGTAAGTGAGCATTC-3′, antisense: 5′-GGCTGTAATCTTCCGAGTC-3′), and *PUMA* (sense: 5′-GCGGAGACAAGAAGAGCAG-3′, antisense: 5′-AGGAGTCCCATGAAGAGATTG-3′). The reference gene was *Ywhaz* (sense: 5′-CCTCAACTTCTCTGTGTTCTATT-3′, antisense: 5′-ACGACTCTTCACTTAATGTATCAA-3′).^51^ The relative expression levels of mRNAs were calculated by the method of geometric averaging of multiple internal control genes.

### Mitochondrial DNA copy number measurement

Total DNA isolation and qRT-PCR were described previously.^37^ The nuclear gene was *Ywhaz* (sense: 5′-GAGGAAGAATCGTGAGTTAGTT-3′, antisense: 5′-TGGTGATGGTTGAGACAGA-3′), and the mitochondrial gene was *ND4* (sense: 5′-CCTCAGTTAGCCACATAGC-3′, antisense: 5′-GATTCGTTCGTAGTTGGAGTT-3′). The relative mtDNA level was calculated based on the threshold cycle (Ct) as 2-Δ (ΔCt).

### Cell counting

The AIF-labeled or Fluoro-Jade-labeled cells were counted in the border zone of cortical infarcts at × 400 magnification. Three sections were counted, each of them 50 sections apart, and expressed as the average number of cells per visual field. The numbers of BrdU-labeled or BLBP-labeled cells were counted in every 50th section throughout the granule cell layer and the subgranular zone (for BrdU) or the subgranular zone only (for BLBP) using stereology microscopy (StereoInvestigator, MicroBrightField Inc., Magdeburg, Germany), and at least six sections were counted from each sample. For the phenotype of BrdU-labeled cells, at least 50 BrdU-positive cells in the dentate gyrus were counted using a confocal laser scanning microscope (Leica TCS SP, Heidelberg, Germany), and the ratio of BrdU/NeuN or BrdU/GFAP double-labeled cells was calculated for each sample. The total number of newborn neurons (BrdU^+^/NeuN^+^) and astrocytes (BrdU^+^/GFAP^+^) in each sample was calculated based on the number of BrdU-positive cells and the ratio of double labeling. All counting was carried out by investigators blinded to the group assignment.

### Statistical analysis

All statistical analyses were performed with SPSS 22.0(Armonk, NY, USA), and all data are expressed as means±S.E.M. Student’s unpaired *t*-test was used to compare the numbers of positive cells and the amount of tissue loss. Ratios were compared using the Mann–Whitney *U*-test. Two-way ANOVA followed by a Bonferroni *post hoc* test was used for multiple comparison correction of data from more than two groups. Significance was assumed when *P*<0.05.
